# Routine use of low-dose glucarpidase following high-dose methotrexate in adult patients with CNS lymphoma: an open-label, multi-center phase I study

**DOI:** 10.1186/s12885-021-09164-x

**Published:** 2022-01-13

**Authors:** Lauren R. Schaff, Mina Lobbous, Dean Carlow, Ryan Schofield, Igor T. Gavrilovic, Alexandra M. Miller, Jacqueline B. Stone, Anna F. Piotrowski, Ugur Sener, Anna Skakodub, Edward P. Acosta, Kevin J. Ryan, Ingo K. Mellinghoff, Lisa M. DeAngelis, Louis B. Nabors, Christian Grommes

**Affiliations:** 1 Memorial Sloan Kettering Cancer Center, Department of Neurology, 1275 York Avenue New York, NY, 10065 New York, NY USA; 2 Department of Neurology, Univeristy of Alabama at Birmingham, Birmingham, AL UK; 3 Memorial Sloan Kettering Cancer Center, Department of Laboratory Medicine, NY New York, USA; 4 Division of Clinical Pharmacology, University of Alabama at Birmingham, Birmingham, AL UK

**Keywords:** CNS lymphoma, Methotrexate, Glucarpidase

## Abstract

**Background:**

High-dose methotrexate (HD-MTX) has broad use in the treatment of central nervous system (CNS) malignancies but confers significant toxicity without inpatient hydration and monitoring. Glucarpidase is a bacterial recombinant enzyme dosed at 50 units (u)/kg, resulting in rapid systemic MTX clearance. The aim of this study was to demonstrate feasibility of low-dose glucarpidase to facilitate MTX clearance in patients with CNS lymphoma (CNSL).

**Methods:**

Eight CNSL patients received HD-MTX 3 or 6 g/m^2^ and glucarpidase 2000 or 1000u 24 h later. Treatments repeated every 2 weeks up to 8 cycles.

**Results:**

Fifty-five treatments were administered. Glucarpidase 2000u yielded > 95% reduction in plasma MTX within 15 min following 33/34 doses (97.1%) and glucarpidase 1000u yielded > 95% reduction following 15/20 doses (75%). Anti-glucarpidase antibodies developed in 4 patients and were associated with MTX rebound. In CSF, glucarpidase was not detected and MTX levels remained cytotoxic after 1 (3299.5 nmol/L, *n* = 8) and 6 h (1254.7 nmol/L, *n* = 7). Treatment was safe and well-tolerated. Radiographic responses in 6 of 8 patients (75%) were as expected following MTX-based therapy.

**Conclusions:**

This study demonstrates feasibility of planned-use low-dose glucarpidase for MTX clearance and supports the hypothesis that glucarpidase does not impact MTX efficacy in the CNS.

**Clinical trial registration:**

NCT03684980 (Registration date 26/09/2018).

## Introduction

Central nervous system lymphoma (CNSL) is an aggressive but treatable malignancy. High-dose methotrexate (HD-MTX) ≥ 3 g/m^2^ is the backbone of the standard-of-care first-line treatment [1], yielding response rates of 50–90%, allowing for implementation of potentially curative consolidation strategies [2–5]. MTX is administered in the inpatient setting with aggressive intravenous hydration and close monitoring of urine pH, renal function, and MTX levels to prevent potentially life-threatening toxicity such as renal failure or pneumonitis.

Glucarpidase is a bacterial recombinant enzyme that cleaves MTX to inactive byproducts glutamate and 4-deoxy-4-amino-N10-methylpteroic acid (DAMPA), rapidly reducing plasma MTX levels > 95% [6, 7]. It is approved by the Food and Drug Administration for use in patients with MTX toxicity and renal failure at a dose of 50 units (u)/kg. Limited retrospective data suggest lower, flat doses of glucarpidase may be equally efficacious [8–10] but prospective dose-finding studies have not been performed. At a size of 83 kDa, glucarpidase is not known to penetrate the blood brain barrier or reduce MTX concentrations in the cerebrospinal fluid (CSF) [11]. Glucarpidase is immunogenic and the development of anti-glucarpidase antibodies has been described but to date, their clinical impact is unknown [12] and efficacy of repeated doses of glucarpidase is unclear.

The immediate clearance of MTX by glucarpidase could have several clinically relevant benefits, including potential reduction of MTX toxicity, prevention of MTX dose-reduction and delay, and abbreviated hospital stay or even outpatient MTX administration. In this prospective study, we explore whether planned-use low-dose glucarpidase (defined as flat-dose glucarpidase 1000 or 2000u) can effectively clear MTX from the plasma without significantly affecting CSF concentrations and whether it can continue to effectively clear MTX from the plasma after repeated doses, throughout a patient’s treatment course. We follow plasma and CSF MTX concentrations as well as the development of anti-glucarpidase antibodies. Finally, we describe safety and clinical efficacy of MTX administered with planned-use low-dose glucarpidase.

## Methods

### Study design and treatment

This was a phase 1, investigator-initiated clinical study of HD-MTX followed by planned-use low-dose glucarpidase for patients with newly diagnosed or relapsed/refractory primary (PCNSL) or secondary CNSL (SCNSL) isolated to the central nervous system. The study was conducted at Memorial Sloan Kettering Cancer Center and the University of Alabama at Birmingham. The study was approved by the institutional review board at each participating institution. All accrued patients provided written informed consent. This trial was registered at www.clinicaltrials.gov as NCT03684980 26/09/2018. This study adheres to CONSORT guidelines.

The primary objective of the study was to determine the ability of planned-use low-dose glucarpidase (2000u and 1000u) to routinely and repeatedly result in significant reduction of plasma MTX levels (> 95% reduction in 6 h) when administered 24 h following MTX. Secondary objectives included MTX pharmacokinetics in the blood and CSF, development of anti-glucarpidase antibodies, safety profile, overall response rate (ORR) (defined as the proportion of subjects with complete response (CR) or partial response (PR)), progression-free survival (PFS) and overall survival (OS). Evaluation of treatment response followed the International Primary CNS Lymphoma Collaborative Group (IPCG) guidelines [13] and occurred after cycles 4 and 8. Response to treatment was assessed in all CNS compartments using MRI imaging and CSF cytology as well as ophthalmologic examination in cases of ocular involvement. Adverse events were graded using the National Cancer Institute (NCI) Common Terminology Criteria for Adverse Events (CTCAE) v.5.0. The toxicity profile was defined in a descriptive manner by documenting all the adverse events at least possibly related to treatment. All patients who received at least one dose of MTX were considered evaluable for toxicity.

Baseline staging to assess disease burden followed the IPCG guidelines [13] and included brain magnetic resonance imaging (MRI), total spine MRI, CSF collection, ophthalmologic examination, and whole-body positron emission tomography (PET). In the absence of a PET scan, a computed tomography (CT) image of the chest, abdomen, pelvis with bone marrow biopsy and testicular ultrasound for men were also acceptable.

Patients were planned for 8 cycles of intravenous MTX. Cycles were 14 days long. Patients were enrolled in two cohorts, filled sequentially; Cohort A (MTX 3.0 g/m^2^) and Cohort B (MTX 6.0 g/m^2^). Three patients were planned for each cohort. Glucarpidase was supplied by BTG, private limited company (London, UK) and was administered 24 (+/− 2) hours following start of MTX. Patients received glucarpidase 2000u during cycles 1–4 of treatment and glucarpidase 1000u during cycles 5–8. In the case of an inadequate response to glucarpidase 1000u, a dose increase back to 2000u was allowed. All patients received rituximab 500 mg/m^2^ with each cycle. MTX was administered intravenously over 2 h.

For the purposes of this study, “low-dose” glucarpidase was defined as the flat doses of 1000 or 2000u. These doses were selected based on previously published retrospective experience [8–10] and the package size of the product. Glucarpidase is dispensed in 1000u vials.

Patients received standard pre- and post-treatment hydration and monitoring of urinary alkalization in the inpatient setting per institutional guidelines. All patients received leucovorin 25 mg orally every 6 h approximately 26 h after start of MTX. Leucovorin was continued 72 h or until MTX concentrations reached ≤100 nmol/L. Intravenous leucovorin was to be added for toxic MTX levels. Filgrastim was permitted at the discretion of the treating physician.

Plasma samples were collected pre-glucarpidase and 15 min, 1 h, 6 h, and every 24 h post-glucarpidase until MTX clearance. CSF samples were collected through lumbar puncture 6 h post-glucarpidase (cycle 1), 1 h post-glucarpidase (cycle 2) and pre-glucarpidase (cycle 5). Patients were discharged when plasma MTX concentrations were ≤ 100 nmol/L.

### Eligibility

We enrolled newly diagnosed, relapsed, or refractory patients with histologically confirmed B-cell non-Hodgkin lymphoma involving the brain, spinal cord, and/or leptomeningeal space. Patients with secondary CNSL were eligible if the central nervous system was their only site of disease at the time of enrollment. Patients with parenchymal lesions were required to have unequivocal evidence of disease progression on imaging 28 days prior to study registration. Patients with leptomeningeal disease only were required to have positive CSF cytology and/or imaging findings consistent with CSF disease 28 days prior to enrollment. There was no limit on the number of prior treatments or relapses allowed.

All patients were at least 18 years old with minimum Karnofsky Performance Status (KPS) of 50. Patients were required to have adequate bone marrow and organ function defined as absolute neutrophil count ≥1 × 10^9^/L, platelets ≥100 × 10^9^/L, hemoglobin ≥8 g/dL, international normalized ratio ≤ 1.5 and PTT ≤ 1.5 times the upper limit of normal (ULN), alanine aminotransferase (ALT) and aspartate aminotransferase (AST) ≤ 3 times the ULN, bilirubin ≤1.5 times the ULN and creatinine clearance ≥60 mL/mi calculated by the Cockcroft-Gault equation.

Exclusion criteria included prior chemotherapy or targeted anticancer therapy administered within 5 half-lives of study initiation, prior RT within 28 days of treatment start, weight < 40 kg, known infection with human immunodeficiency virus or active/chronic hepatitis B or C virus, evidence of large pleural or ascitic fluid collection, or severe medical co-morbidity.

Criteria for study removal included progression of disease (PD), development of an intercurrent illness preventing administration of study treatment, patient or physician preference, pregnancy, patient non-compliance, study completion, or patient death. Patients who did not complete at least 6 cycles of MTX with planned-use low-dose glucarpidase were replaced so that the safety and efficacy (the ability to clear MTX from the plasma) of repeated glucarpidase administrations in a single patient could be explored.

### Assessments

#### Plasma and CSF MTX pharmacokinetics

Plasma samples were collected pre-glucarpidase and at 15 min, 1 h, 6 h, and every 24 h post-glucarpidase until MTX clearance (≤100 nmol/L). MTX immunoassay was performed in addition to high-pressure liquid chromatography – mass spectrometry (LC-MS/MS) to differentiate between MTX and DAMPA following glucarpidase [14, 15]. Plasma samples were collected for analysis of anti-glucarpidase antibodies on Day 1 of each cycle.

CSF samples were collected through lumbar puncture 6 h post-glucarpidase (cycle 1), 1 h post-glucarpidase (cycle 2) and pre-glucarpidase (cycle 5) and underwent testing with LC-MS/MS to determine MTX and DAMPA concentrations. Detection of glucarpidase in CSF was performed by Eurofins Pharma Bioanalyses Services UK employing quantitative sandwich ELISA with Rabbit anti-glucarpidase capture reagent.

### Statistical analyses

Descriptive statistics including medians, standard deviations, and means for continuous variables and proportions for discrete variables, were used to summarize the findings in each cohort. The Kaplan-Meier method was used for time-to-event analysis. PFS was calculated from trial registration until disease progression, last clinical assessment, or death, whichever came first. Progression and deaths were considered events in the PFS analysis. OS was calculated from trial registration until death or last follow-up. Deaths were considered events in OS analysis.

## Results

### Patient characteristics

An enrollment of 6 patients was planned however, a total of 8 patients were enrolled between 19/11/2018 and 18/9/2019 (7 PCNSL, 1 SCNSL); because 2 of the originally planned 6 patients did not complete at least 6 cycles of MTX, a requirement of this study. They were subsequently replaced with 2 additional patients. Four patients were treated with MTX 3 g/m^2^ and 4 with 6 g/m^2^. Median follow up was 15.2 months. Six (75%) patients were men and 2 (25%) were women (Table 1). The median age was 70 (range, 57–78) and median KPS was 70 (range, 50–100). All 7 patients with PCNSL were newly diagnosed. The patient with SCNSL was receiving 4th line treatment (last CNS-directed therapy within two weeks of study enrollment; last MTX dose was administered 12 months prior to study enrollment). None of the patients had evidence of ocular disease on slit lamp examination. Three patients had confirmed or suspected leptomeningeal disease.

**Table 1:** Patient Characteristics

Sex, No. (%)	
Men	6 (75%)
Women	2 (25%)
**Median age, years (range)**	70 (57–78)
**KPS, (range)**	70 (50–100)
**Location, No. (%)**	
Primary	7 (87.5%)
Secondary	1 (12.5%)
**Disease Status**	
Newly diagnosed	7 (87.5%)
Recurrent	1 (12.5%)
**Leptomeningeal disease**	3 (37.5%)
**Ocular disease**	0 (0%)
**Median Prior Regimens, No. (range)**	0 (0–3)
**MTX 3 g/m**^**2**^**(patients)**	4
Glucarpidase 2000u	17 doses
Glucarpidase 1000u	11 doses
**MTX 6 g/m**^**2**^**(patients)**	4
Glucarpidase 2000u	18 doses
Glucarpidase 1000u	9 doses

Eight doses of MTX were planned for each patient. Only 55 of the 64 planned doses of MTX were given due to disease progression and withdrawal from study of 3 patients. One patient (Cohort B; #6) withdrew from study after 6 cycles of MTX when MRI demonstrated complete radiographic response without clinical improvement; one patient (Cohort A; #3) withdrew after 4 cycles due to lack of clinical or radiographic improvement; and one patient (Cohort B; #5) experienced progressive disease (PD) after 5 cycles. Post-glucarpidase MTX levels are available following 54 doses of MTX. Fifty-two doses were at the planned 3 g/m^2^ (28) or 6 g/m^2^ (24). One patient was dose-reduced from 6 to 4 g/m^2^ for two MTX treatments following a grade 2 creatinine increase. Five patients (62.5%) received all eight planned cycles of MTX.

### Plasma MTX reduction

**Fig. 1:**
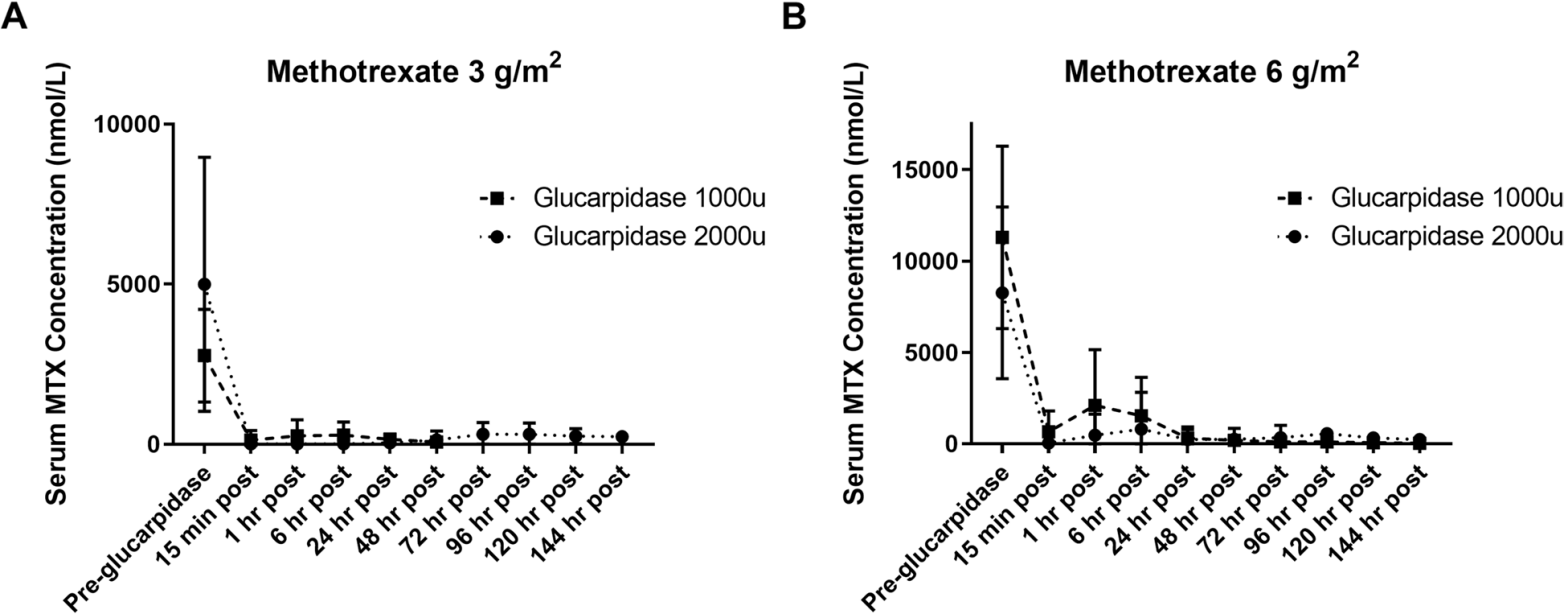
Serum methotrexate (MTX) concentrations with glucarpidase. Serum methotrexate concentrations following administration of MTX 3 g/m^2^ (A) and 6 g/m^2^ (B) followed by glucarpidase 1000 or 2000u

Glucarpidase 2000u or 1000u was administered 24 ± 2 h after MTX. Thirty-five doses at 2000u and 20 at 1000u were administered. Due to sample loss (Patient 6, cycle 1), plasma MTX data is available following 34 doses of 2000u. Plasma MTX concentrations were reduced from a median of 4960 nmol/L to 22 nmol/L (99.7%) within 15 min of glucarpidase administration. A reduction in plasma MTX concentration of at least 95% was seen following 33/34 (97.1%) doses of glucarpidase 2000u (93.1 to > 99%) and following 15/20 (75%) doses of glucarpidase 1000u (72.6 to > 99%) (Fig. 1). Formation of DAMPA peaked 15 min following glucarpidase administration and cleared at a median of 24 h, following first order kinetics.

### Anti-glucarpidase antibody formation

Four patients (two in each cohort) (#1, #4, #6, #7) developed anti-glucarpidase antibodies. Antibodies were detected after cycle 2 (2) or cycle 3 (2) of treatment and appeared independent of patient steroid use. In all, anti-glucarpidase antibodies were detected in 21/52 plasma samples (40.4%) and were determined or presumed neutralizing (based on prior and subsequent data from the same patient) in all but 1 of these samples (95.2%).

**Fig. 2:**
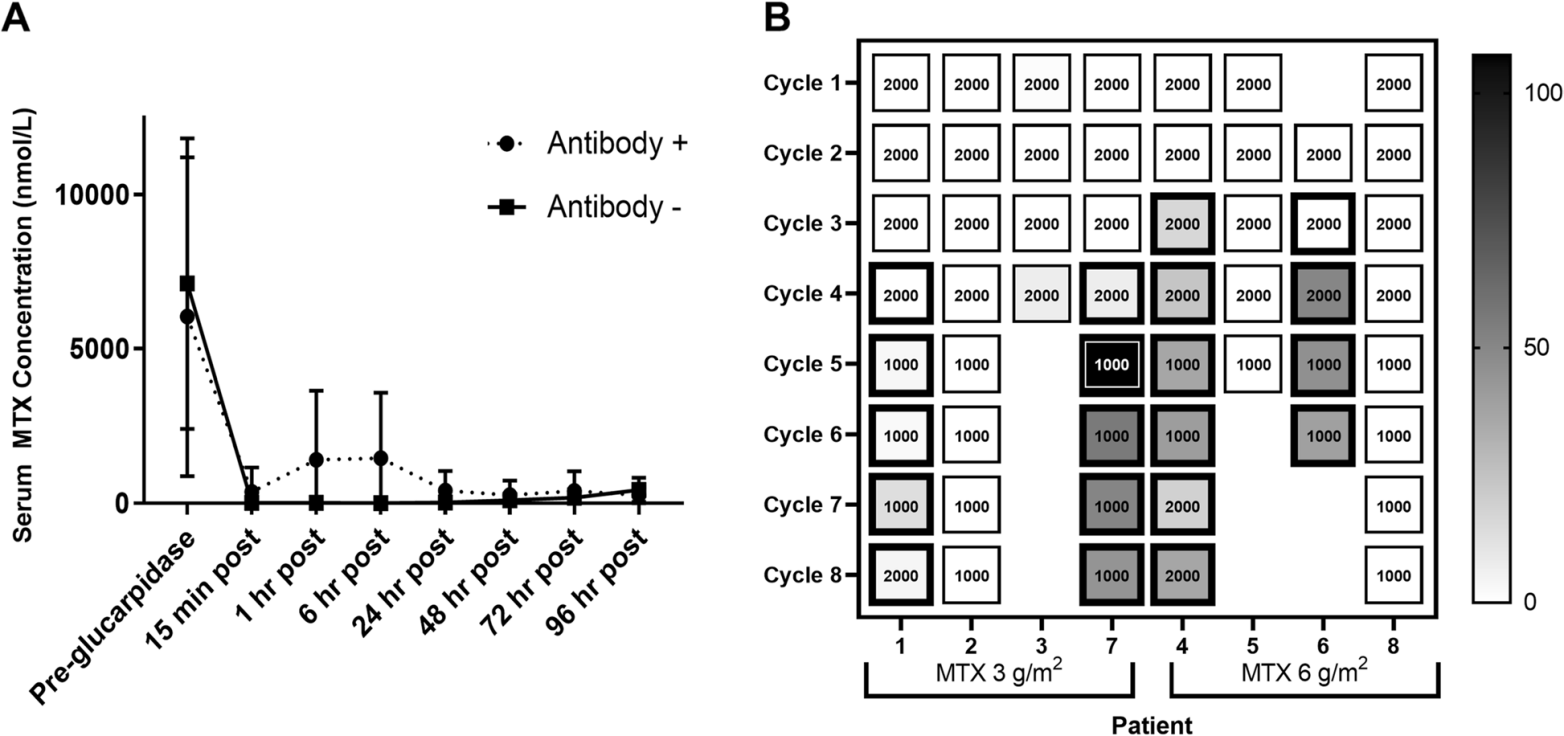
Effect of anti-glucarpidase antibodies on serum methotrexate (MTX concentrations after glucarpidase. (A) MTX concentrations pre- and post-glucarpidase in patients with and without anti-glucarpidase antibody formation. Blue line reflects MTX concentrations in patient with anti-glucarpidase antibodies and red, without. (B) Heat map of MTX rebound data as % of pre-glucarpidase MTX concentration. Cell label represents dose of glucarpidase, either 2000 or 1000 units. Thickened border indicates presence of neutralizing anti-glucarpidase antibody. Patient 6 cycle 1 results not available due to sample loss

In the absence of neutralizing antibodies, glucarpidase reduced plasma MTX > 98% within 15 min (32/32). When neutralizing antibodies were present (20), glucarpidase resulted in a median reduction of 98.9% (range, 72.6 - > 99%) of plasma MTX concentration though MTX level failed to be reduced > 95% after 6 MTX doses in patient #4 (1), #6 (2), and #7 (3)(30%) (Fig. 2A).

A MTX rebound (rise in MTX plasma concentration to > 100 nmol/L after initial clearance or an increase in plasma MTX concentration after initial reduction) occurred following 20/54 (37%) doses of glucarpidase. Eighteen of these cases were in the setting of confirmed or suspected neutralizing anti-glucarpidase antibody. Twice, rebound occurred in the absence of antibody formation (Patient #3). When antibody was present, MTX concentrations rose from a median of 75 nmol/L (< 5–2872.9 nmol/L) to 651.6 nmol/L (0–8201 nmol/L) or to 30.2% of the pre-glucarpidase MTX concentration (0–107.5%). Rebound was first detected at a median of 1 h after glucarpidase administration (1–24 h) and resolved by 48 h (24–120 h). In the patient lacking neutralizing antibodies (#3), rebound was first noted 24 and 48 h after glucarpidase and peaked at a median of 489.5 nmol/L (162–817 nmol/L), or 4.2% (1.2–7.1%) of the pre-glucarpidase MTX concentration. Rebound was associated with a grade 2 creatinine increase in 3 cycles [Patient #3 (2) and #6 (1)]. In Patient #3, lacking anti-glucarpidase antibodies, the creatinine increase occurred during cycles 1 and 4 and preceded the development of MTX rebound. In Patient #6, creatinine had increased from 0.6 to 1.3 mg/dL prior to glucarpidase administration during cycle 4 and peaked at 2.1 mg/dL 96 h after MTX.

Patients who developed neutralizing antibodies appeared to have progressive difficulty clearing MTX following glucarpidase and developed more profound rebound over time. In response to rebound, glucarpidase dose was increased from planned 1000u to 2000u for three cycles (Patient #1 and #4). In these instances, the increased glucarpidase dose appeared to result in improved MTX clearance and less rebound (Fig. 2B).

### Cerebrospinal fluid

**Fig. 3:**
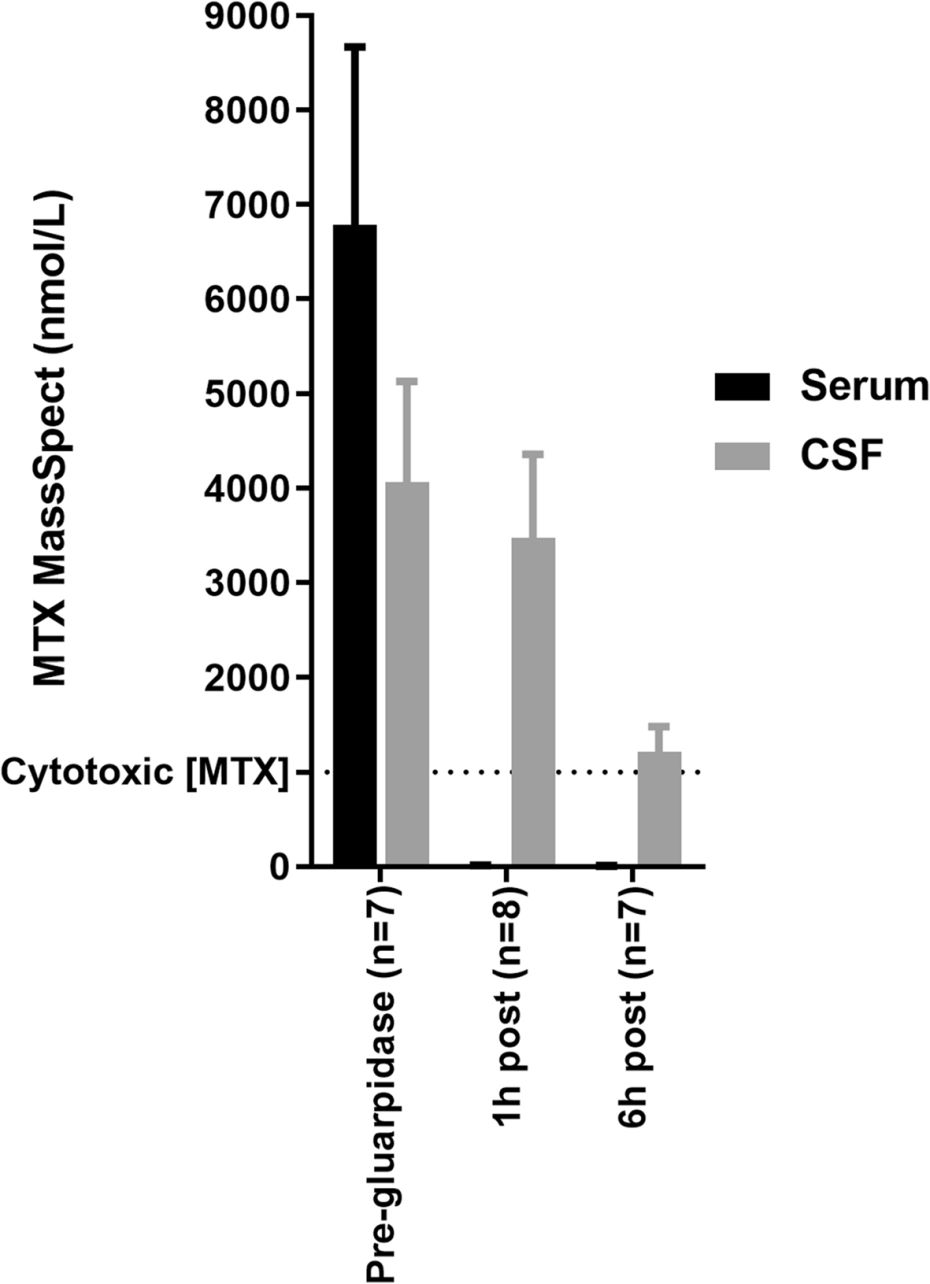
Methotrexate (MTX) concentrations in cerebrospinal fluid (CSF). Methotrexate concentrations in the serum and cerebrospinal fluid (CSF) pre-, 1 h post-, and 6 h post-glucarpidase. Red bar reflects serum values, blue, cerebrospinal fluid

Patients underwent lumbar puncture pre-glucarpidase, (*n* = 7), 1 h (*n* = 8), and 6 h post-glucarpidase administration (n = 7). Pre-glucarpidase median MTX concentration was 3305.8 (1274.7–8751.1 nmol/L). MTX concentrations were 3299.5 (301–6737.4 nmol/L) and 1254.7 (500.4–2293.3 nmol/L) at 1 and 6 h post-glucarpidase, respectively (Fig. 3). Median concentration of DAMPA was 0 (0–45.5 nmol/L) pre-glucarpidase and 163.7 (0–436.2 nmol/L) and 1173.9 (233.9–2352.7 nmol/L) at 1 and 6 h post-glucarpidase.

Seventeen CSF samples from 6 patients were analyzed for the presence of glucarpidase which was not detected in any of the samples.

### Toxicity

Toxicities are outlined in Table 2. There were no grade 3 or higher toxicities associated with glucarpidase. We observed 21 grade 3 events at least possibly attributed to MTX (most frequent: 10 lymphopenia, 5 anemia, 2 hypokalemia). The most common adverse events were lymphopenia (32), anemia (27), increased AST (20) and ALT (15), increased bilirubin (14), hypoalbuminemia (12), leukopenia (12), nausea (12), and hypokalemia (10). Grade 1 and 2 creatinine increases were noted with 5 cycles of treatment between Patient #3 (4) and Patient #6 (1). In all cases, creatinine elevation began within the first 24 h of MTX, prior to glucarpidase administration.

**Table 2:** Adverse events

	Grade 1	Grade 2	Grade 3	Grade 4
**Adverse Reactions at least possibly related to glucarpidase (All)**				
Abdominal pain	–	1 (2)	–	–
Dyspnea	–	1 (2)	–	–
Fecal incontinence	1 (2)	–	–	–
Flushed face	1 (2)	–	–	–
Headache	–	1 (2)	–	–
Nausea	3 (5)	–	–	–
**Adverse Reactions at least possibly related to methotrexate (All grade 3 and higher, most common > 10% grade 1–2)**				
Alanine aminotransferase increased	12 (22)	2 (4)	1 (2)	–
Anemia	10 (18)	12 (22)	5 (9)	–
Aspartate aminotransferase increased	17 (31)	2 (4)	1 (2)	–
Blood billirubin increased	11 (20)	3 (5)	–	–
Creatinine increased	2 (4)	3 (5)	–	–
Hypoalbuminemia	10 (18)	2 (4)	–	–
Hypokalemia	7 (13)	1 (2)	2 (4)	–
Lung infection	–	–	1 (2)	–
Lymphocyte count decreased	7 (13)	15 (27)	10 (18)	–
Nausea	8 (15)	4 (7)	–	–
White blood cell count decreased	8 (15)	3 (5)	1 (2)	–

-, not observed

Methotrexate was dose-reduced to 4 g/m^2^ for one patient in Cohort B (Patient #6) following a grade 2 creatinine increase.

There were no grade 4 adverse events attributed to treatment.

### Response

**Fig. 4:**
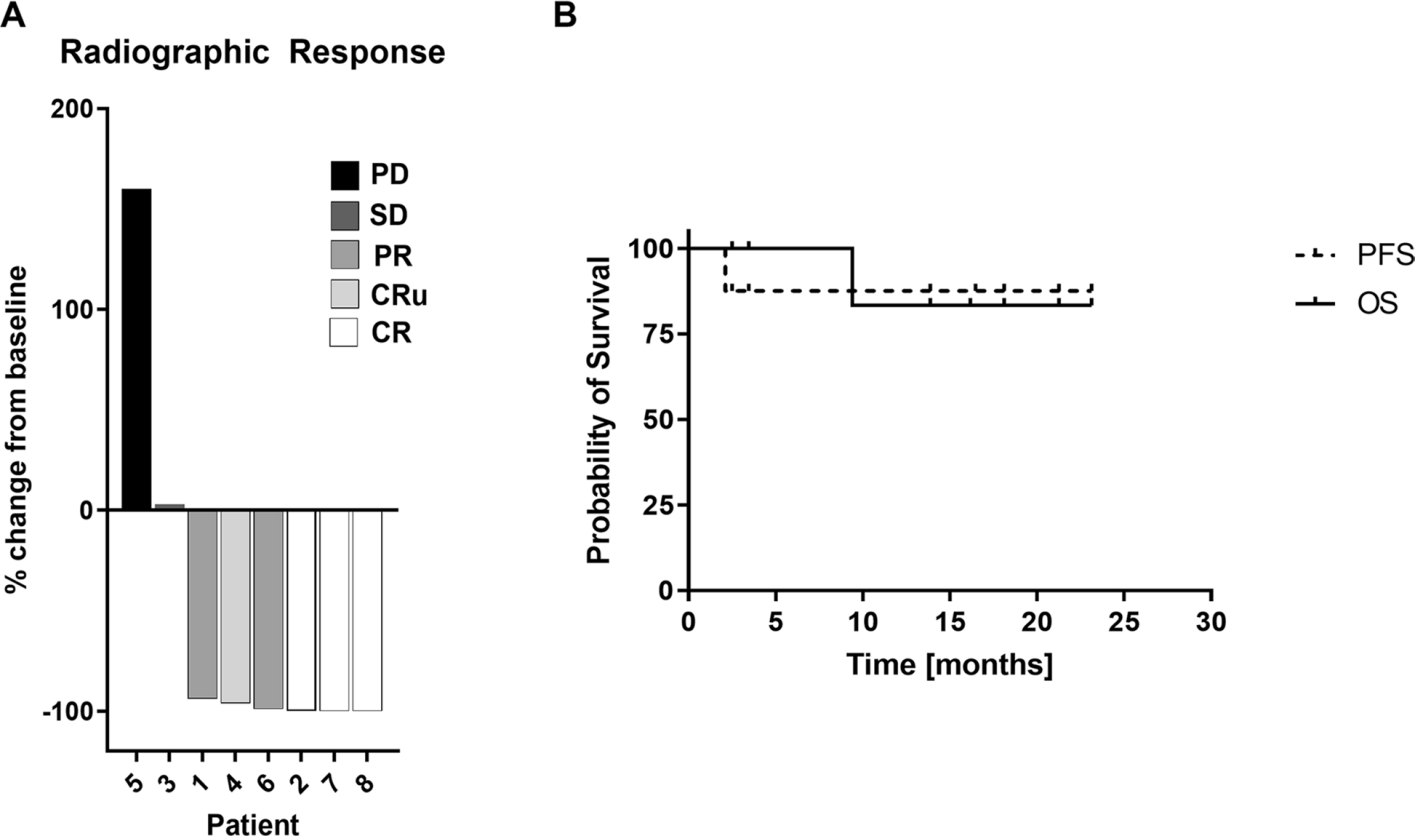
Clinical response in patients treated with methotrexate (MTX) and glucarpidase. **(A)** Radiographic response to MTX in combination with glucarpidase, assessed using International PCNSL Collaborative Group Guidelines. Displayed is the change in target lesion diameter from baseline (%) by magnetic resonance imaging. Negative values indicate tumor shrinkage. Red indicates progression of disease (PD); orange, stable disease (SD); green, partial response (PR); purple, unconfirmed complete response (CRu); blue, complete response (CR) (**B)** Kaplan-Meier curves reflecting progression-free (PFS) and overall survival (OS) for study patients

Radiographic responses were seen in 6 patients (75%) with one stable disease and one with disease progression; responses included complete response (CR) in 3, unconfirmed complete response (CRu) in 2, and partial response (PR) in 1 (Fig. 4A). Patient #6 elected to forego further tumor-directed therapy after 6 cycles of MTX despite radiographic CRu. One patient had minimal residual enhancement after 8 cycles of MTX that further resolved after consolidation therapy, thus response to MTX was considered CRu.

One patient had stable non-enhancing disease after 4 cycles of MTX and withdrew from study, declining further tumor-directed therapy due to lack of clinical benefit. The patient receiving 4th line treatment had progressive disease (PD) after five cycles of MTX.

All patients with suspected or confirmed leptomeningeal involvement had documented clearance of CSF by cytology and flow cytometry following induction therapy.

In total, five of the six patients with radiographic response elected to proceed to consolidation with either high-dose cytarabine alone (2) or high-dose chemotherapy followed by autologous stem cell transplant (3). All 5 patients with radiographic response followed by consolidation remain alive and progression-free. Median PFS and OS were not reached (Fig. 4B) and a median follow up of 16 months.

## Discussion

Our study demonstrates the feasibility of planned-use low-dose glucarpidase for rapid plasma MTX reduction in patients with CNSL. While the role of glucarpidase in MTX toxicity has been well established at the standard dose of 50 u/kg, this study addresses previously unanswered questions regarding efficacy of low-dose glucarpidase, potential role of repeated empiric administrations, and the impact of anti-glucarpidase antibodies. In this study, we selected flat doses of glucarpidase as it functions within the blood stream, the volume of which is largely independent of weight.

Median MTX reduction following low-dose glucarpidase was 99.7% with all doses precipitating at least a 70% reduction in plasma MTX concentrations. While transient elevations in MTX concentrations were seen following 37% of glucarpidase doses, rebound was short lived and not clearly of clinical significance.

Anti-glucarpidase antibody development was detected in 50% of patients in this study. In almost all cases, antibodies exhibited neutralizing activity. Unlike in prior studies, we did not see any antibody formation after a single dose of glucarpidase [9, 12], possibly as a result of the lower dose used in this study. Antibody formation occurred after either two or three glucarpidase doses and 4 patients never developed antibodies despite a median of 6.5 doses of glucarpidase. There was no clear association between steroid use and antibody formation though this analysis is limited due to small sample size. While the efficacy of glucarpidase was modestly affected in the presence of neutralizing antibodies, a significant reduction in plasma MTX concentrations was still achieved and this impact was mitigated by escalating the glucarpidase dose in subsequent MTX cycles. MTX rebound was more profound in patients with anti-glucarpidase antibodies. We propose this is due to redistribution of MTX from the tissues back into the vasculature after neutralization of glucarpidase, which typically confers a half-life of 5.6 h [16]. Rebound was also seen in the setting of acute kidney injury that preceded glucarpidase.

Overall, this treatment regimen was well tolerated. An earlier study of planned-use full-dose glucarpidase (50 u/kg) was closed to accrual due to dose-limiting toxicities in two patients: an acute kidney injury and grade 2 infusion reaction to glucarpidase [17]. While we did see a grade 1 or 2 creatinine rise following 5 of 55 treatments (9%), the creatinine elevation began prior to glucarpidase administration and was attributed to methotrexate. This suggests nephrotoxicity from MTX is an early event, beginning within 24 h of administration. We hypothesize that the rapid reduction of MTX by glucarpidase may have prevented a more serious nephrotoxicity in these patients. Glucarpidase itself was well tolerated. The reported grade 1 and 2 reactions occurred in ≤5% of administrations and did not appear associated with development of anti-glucarpidase antibodies, a finding similarly observed in a retrospective study [12].

Efficacy of MTX did not appear to be affected by planned-use glucarpidase. Radiographic response was seen in 75% of enrolled patients including 86% of newly diagnosed patients, concordant with previously reported responses to MTX [2–5]. The one patient in our study with PD was heavily pre-treated, receiving fourth line therapy, and had previously received MTX. PFS and OS did not appear to be negatively impacted by the use of glucarpidase though we acknowledge this is a small sample size and as such, survival data should be interpreted with caution. Glucarpidase does not appear to affect MTX efficacy in the leptomeningeal space. It is not known to cross the blood-brain barrier [11] and in our study, was not detected in 17 of 17 CSF samples. An earlier study of MTX pharmacokinetics after glucarpidase suggests MTX concentrations reduce slowly in the CSF, following first-order kinetics [11]. This is consistent with data suggesting MTX efflux from the CNS is independent of plasma drug concentrations and should not be affected by plasma clearance [18]. As seen in a prior study, DAMPA concentrations in CSF increased slowly over time, suggesting diffusion from the plasma as opposed to rapid formation generated by MTX hydrolysis in the CSF [11]. In the present study, MTX concentrations remained cytotoxic in the CSF at 1 and 6 h after glucarpidase administration (25 and 30 h after MTX infusion) and all patients with suspected or confirmed leptomeningeal involvement at diagnosis had resolution of CSF disease.

While this study successfully demonstrates that low-dose glucarpidase is effective for rapid plasma MTX clearance, a full exploration of the 1000u dose level was limited by study design. While the 2000u dose may appear more effective in preventing rebound (Fig. 1) these data are confounded by the study design as well as timing of antibody development. Since all patients received the 2000u dose of glucarpidase during the first 4 cycles of treatment, there are more data points for this dose level (34 vs 20). Additionally, a smaller percentage of these doses were administered in the setting of pre-formed anti-glucarpidase antibody (26.5% vs 55%). In the absence of antibody, the 1000u dose was effective in rapidly reducing MTX concentrations and preventing rebound, consistent with retrospective data [10]. In patients who never developed antibodies, the 1000u does remained effective for their remaining cycles of treatment. It is possible that moving forward, even lower doses of glucarpidase may demonstrate effectiveness and perhaps lower doses would be less immunogenic.

Planned-use low-dose glucarpidase appears overall effective for the rapid clearance of plasma MTX. CSF pharmacokinetics and rate of radiographic response suggest MTX efficacy in the CNS is not negatively affected by planned-use glucarpidase administration. Further data are necessary to explore the role and impact of anti-glucarpidase antibody formation as our sample size was small. We believe further studies are justified to explore the use of planned-use glucarpidase to optimize HD-MTX administration.

## Data Availability

The datasets generated during and/or analyzed during the current study are not publicly available but are available from the corresponding author on reasonable request.

## Abbreviations

ALT: Alanine aminotransferase
AST: Aspartate aminotransferase
CNS: Central nervous system
CNSL: Central nervous system lymphoma
CR: Complete response
CRu: Unconfirmed complete response
CT: Computed tomography
CTCAE: Common Terminology Criteria for Adverse Events
CSF: Cerebrospinal fluid
DAMPA: 4-deoxy-4-amino-N10-methylpteroic acid
HD-MTX: High-dose methotrexate
IPCG: International Primary CNS Lymphoma Group
KPS: Karnofsky Performance Status
LC-MS/MS: High-pressure liquid chromatography-mass spectrometry
MRI: Magnetic resonance imaging
MTX: Methotrexate
NCI: National Cancer Institute
ORR: Overall response rate
OS: Overall survival
PCNSL: Primary central nervous system lymphoma
PD: Progression of disease
PET: Positron emission tomography
PFS: Progression-free survival
PR: Partial response
SCNSL: Secondary central nervous system lymphoma
u: Units
ULN: Upper limit of normal
